# An improved cucumber mosaic virus-based vector for efficient decoying of plant microRNAs

**DOI:** 10.1038/srep13178

**Published:** 2015-08-17

**Authors:** Qiansheng Liao, Yifei Tu, John P. Carr, Zhiyou Du

**Affiliations:** 1 College of Life Sciences, Zhejiang Sci-Tech University, Hangzhou 310018, China; 2 Department of Plant Sciences, University of Cambridge, Cambridge CB2 3EA, United Kingdom

## Abstract

We previously devised a cucumber mosaic virus (CMV)-based vector system carrying microRNA target mimic sequences for analysis of microRNA function in *Arabidopsis thaliana*. We describe an improved version in which target mimic cloning is achieved by annealing two partly-overlapping complementary DNA oligonucleotides for insertion into an infectious clone of CMV RNA3 (LS strain) fused to the cauliflower mosaic virus-derived 35S promoter. LS-CMV variants carrying mimic sequences were generated by co-infiltrating plants with *Agrobacterium tumefaciens* cells harboring engineered RNA3 with cells carrying RNA1 and RNA2 infectious clones. The utility of using agroinfection to deliver LS-CMV-derived microRNA target mimic sequences was demonstrated using a miR165/166 target mimic and three solanaceous hosts: *Nicotiana benthamiana*, tobacco (*N. tabacum*), and tomato (*Solanum lycopersicum*). In all three hosts the miR165/166 target mimic induced marked changes in developmental phenotype. Inhibition of miRNA accumulation and increased target mRNA (*HD-ZIP III*) accumulation was demonstrated in tomato. Thus, a CMV-derived target mimic delivered via agroinfection is a simple, cheap and powerful means of launching virus-based miRNA mimics and is likely to be useful for high-throughput investigation of miRNA function in a wide range of plants.

MicroRNAs (miRNA) are short regulatory RNA molecules, ranging in length between 20 and 24 nucleotides that in plants and other eukaryotes control gene expression at the post-transcriptional level^1^. In plants, mature miRNA/miRNA* duplexes are processed from the primary pri-miRNA transcripts of *MIR* genes by the endonuclease activity of Dicer-Like 1 (DCL1)^2^. Mature miRNAs are incorporated into Argonaute (AGO)-containing RNA-induced silencing complexes (RISC) to guide sequence-specific cleavage or translational inhibition of target mRNAs^3^,^4^. In plants, miRNAs play vital roles in regulation of development and in resilience against biotic attack and abiotic stress^5^,^6^,^7^,^8^,^9^,^10^. Although much progress has been made in revealing miRNA functions in model species, such as *Arabidopsis thaliana*, their roles are still incompletely understood, particularly in less intensively studied plants. Thus, development of inexpensive, rapid, high-throughput methods applicable to multiple plant species is needed.

Target mimic (TM) sequences provide a natural regulatory mechanism for miRNA activity. In *A. thaliana*, inhibition of miR399 occurs by interaction with a non-cleavable target mimic sequence in transcripts of the gene *Induced by Phosphate Starvation1* (*IPS1*)^11^. Subsequently, an artificial *IPS1*-based TM technique was developed to investigate miRNA function by constitutive expression of artificial TM sequences in transgenic *A. thaliana* plants^12^,^13^,^14^. Yan *et al.*^15^ reported that transgenic expression of a short tandem target mimic (STTM), composed of two TMs separated by a short spacer sequence was more effective at inactivating miRNA activity than the *IPS1*-based TM.

Compared with constitutive expression in transgenic plants, viral vectors have advantages in terms of rapid production and potentially higher throughput for analysis of miRNA function. Sha *et al.*^16^ utilized the tobacco rattle virus (TRV)-based vector^17^ to express TMs or STTMs to inhibit specific miRNAs in *Nicotiana benthamiana* and tomato (*Solanum lycopersicum*). Subsequently, a similar TRV-based TM expression approach was attempted in *A. thaliana*^18^. However, Sha and colleagues^16^ found that only 20–30% of plants infected with TRV variants expressing either TM or STTM exhibited the expected phenotypes. Independently, we developed a TM vector based on cucumber mosaic virus (CMV) strain LS, in which miRNA TM sequences were introduced at a point in the RNA3 sequence immediately downstream of the coat protein (CP) gene^19^. The efficacy of this CMV-based TM vector was demonstrated well by the efficient inhibition of activity of artificial and endogenous miRNAs in *A. thaliana* and a miR159 TM expressed from the LS-CMV vector (LS-MIM159) was used to show the importance of the corresponding miRNA159 in the evocation of disease symptoms in *A. thaliana* plants by the severe strain, Fny-CMV^19^.

Although CMV has potential advantages over other viruses for application to a wide range of plant species (due to its unparalleled host range)^20^,^21^ we realized that the previous version of the CMV-based vector is not well adapted for time- and cost-effectiveness, since it requires reconstitution of the virus from three infectious cDNA clones by *in vitro* transcription and mechanical inoculation, which is expensive and may be inefficient for some host species. In this work we simplified the procedure by incorporating the virus-derived sequences into T-DNAs, allowing the launch of CMV-derived TM vectors using agroinfection.

## Results and Discussion

**Figure 1:**
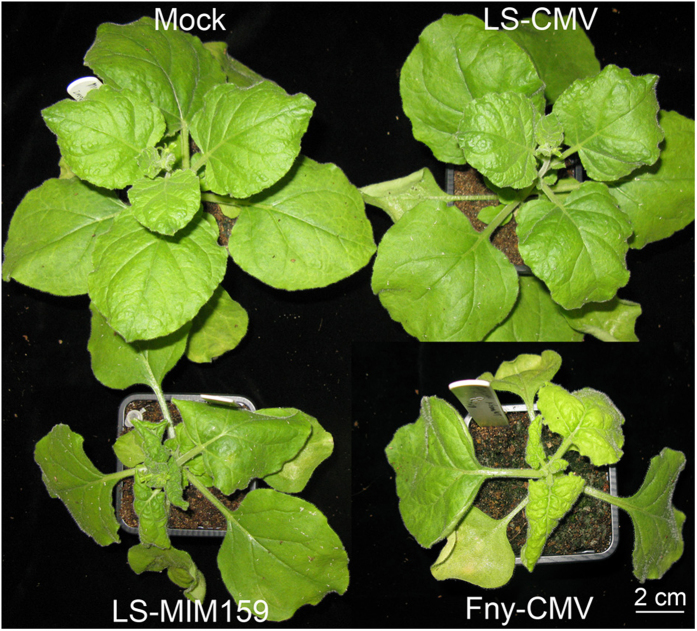
The microRNA miR159 may play a role in disease symptom induction by Fny-CMV in the solanaceous host, *Nicotiana benthamiana.* Appearance of *N. benthamiana* plants 10 days following mechanical inoculation with *in vitro*-synthesized RNA for LS-CMV, LS-MIM159, or Fny-CMV, or mock-inoculation with sterile distilled water.

We investigated the effects of the LS-CMV-derived TM vector in three species of the *Solanaceae*, a large plant Family that includes many important crop species. These hosts were *N. benthamiana*, tobacco and tomato. LS-CMV induces barely discernable symptoms in *A. thaliana* but it has been reported to cause mild systemic symptoms in tobacco and tomato^19^,^22^,^23^. To ensure that the effects of TM expression on plant phenotype could be distinguished from virus-induced symptoms in a solanaceous host we inoculated *N. benthamiana* plants with a mixture of *in vitro*-synthesized LS-CMV RNAs 1 and 2 with RNA3 modified to express a TM for miR159 (LS-MIM159)^19^. Under our growth conditions LS-CMV did not induce discernable symptoms in *N. benthamiana* plants but LS-MIM159 induced severe stunting and deformation of host plants similar to the symptoms induced by the severe strain, Fny-CMV (Fig. 1). The results showed that it is practical to use LS-CMV as a vector for TM sequences in *N. benthamiana*. Furthermore, the results suggest that interference with miR159 activity may have a role in the induction of disease by severe CMV strains in solanaceous hosts, similar to its role in the pathogenesis of a severe strain (Fny-CMV) in *A. thaliana*^19^.

**Figure 2:**
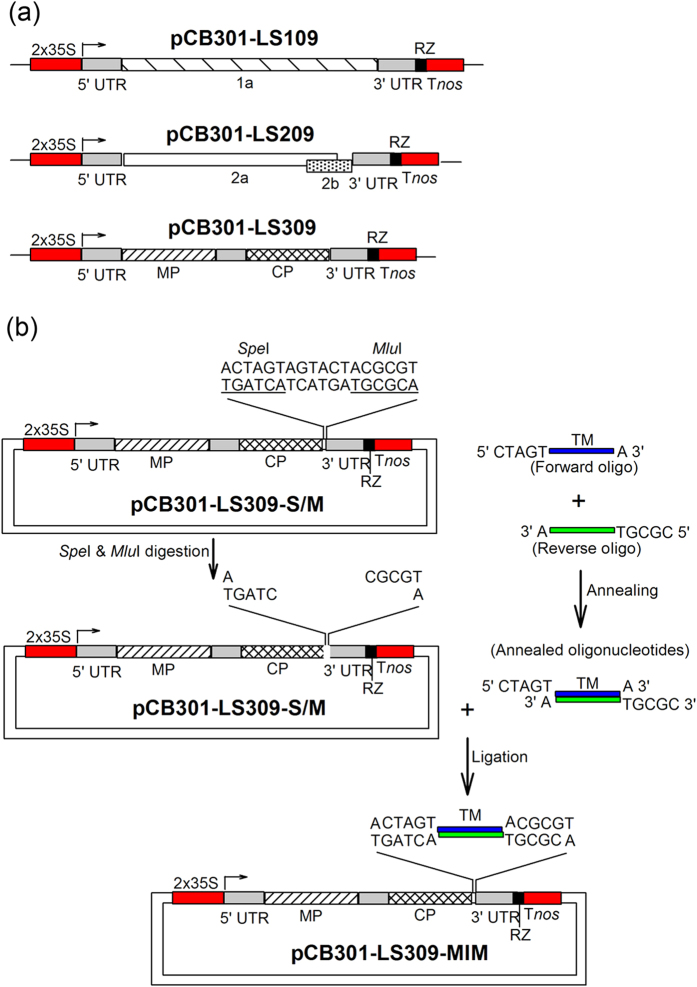
A simplified pathway to generation of CMV-based vectors for expression of miRNA target mimic (TM) sequences in plants. (**a**) Schematic diagram of the infectious clones for LS-CMV genomic RNAs 1–3 inserted into expression cassettes controlled by a double-35S constitutive promoter and nos terminator sequence. (**b**) The steps for cloning a miRNA TM sequence into the infectious clone of LS-CMV RNA3. Two partly overlapping and completely complementary oligonucleotides were used to introduce restriction endonuclease sites *Spe*I and *Mlu*I for cloning inserts between *CP* gene and 3′ untranslated region (UTR) to generate pCB301-LS309-S/M. ‘Forward oligo’ represents TM sequence flanked by 5′ CTAGTA and 3′ A, and ‘Reverse oligo’ represent complementary sequences of TM flanked by 5′ CGCGT and 3′ A. Both oligonucleotides were annealed to produce a duplex with two overhangs, 5′ CTAG and 5′ CGCG, and directly inserted into the pCB301-LS309-S/M vector previously linearized with *Spe*I and *Mlu*I, to generate pCB301-LS309-MIM.

To test the feasibility of launching the CMV-derived TM vector by agroinfection we first constructed T-DNA vectors harboring infectious clones for LS-CMV RNAs1, 2, and 3 that were called, respectively, pCB301-LS109, pCB301-LS209, and pCB301-LS309 (Fig. 2a). The plasmids pCB301-LS309-MIM165/166 and the target mimic control (containing a dummy TM unrelated to any known miRNA target sequence) pCB301-LS309-MIM-CK were also generated using sequences adopted from previous reports^13^,^19^ (Fig. 2b). We selected the miR165/166 TM sequence (MIM165/166) since, when expressed from LS-CMV, it efficiently inhibited miR165/166 activity in wild-type Arabidopsis plants^19^. In addition, a STTM sequence for miR165/166 expressed by a TRV variant launched by agroinfection induced phenotypic changes in *N. benthamiana*^16^. Importantly, the genes encoding miR165/166 and its target transcript *HD-ZIP III* have been evolutionarily conserved^24^, making this system a good model for testing the efficacy of TM technology across multiple plant species.

**Figure 3:**
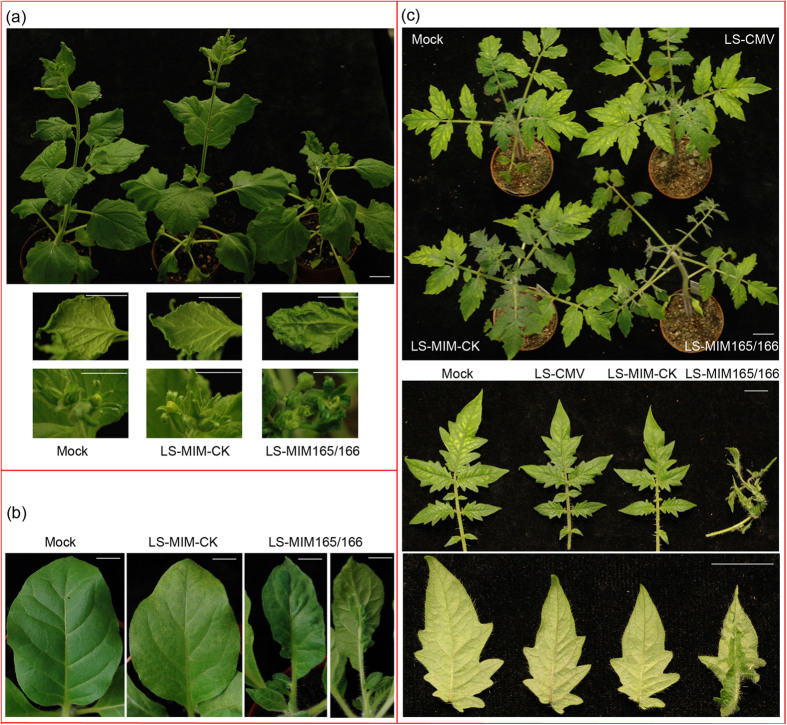
Expression of a miR165/166 target mimic sequence (MIM165/166) in LS-CMV remarkably alters phenotypes of solanaceous species. (**a**,**b**) lower leaves of *Nicotiana benthamiana* and *N. tabacum* (tobacco) plants were infected with LS-MIM-CK or LS-MIM165/166 by agroinfiltration or mock-inoculated with buffer. In (**a**) the upper, middle and lower panels show, respectively, the appearances of whole plants, non-inoculated/systemically-infected upper leaves and developing inflorescences, while in (**b**) non-inoculated/systemically-infected tobacco leaves are shown. (**c**) Tomato (*Solanum lycopersicum*) plants were agroinfected with LS-CMV, LS-MIM-CK or LS-MIM165/166 and upper, middle and lower panels show, respectively, whole plants, upper (non-inoculated/systemically infected) compound leaves, and individual leaflets. ‘Mock’ indicates the plants infiltrated with infiltration solution alone. Plants were photographed 28 days post-infiltration. Scale bars represent 1 cm with exceptions of bars representing 2 cm shown in upper panels of (**a**,**c**).

We agroinfected leaves of plants of *N. benthamiana*, tobacco and tomato with mixtures of *A. tumefaciens* cells transformed with either pCB301-LS109 or pCB301-LS209, together with cells carrying either pCB301-LS309 (containing the wild-type LS-RNA3 sequence) or pCB301-LS309-MIM165/166 (containing the LS-RNA3 sequence modified to carry the miR165/166 TM sequence), or pCB301-LS309-MIM-CK (harboring the dummy TM). At 28 days post-inoculation (dpi), *N. benthamiana* plants agroinfected with the LS-MIM-CK construct displayed no discernable differences in phenotype with the mock-inoculated plants (leaves infiltrated with buffer alone) (Fig. 3a). This is consistent with the weak-to-non-discernable symptoms induced by LS-CMV in *N. benthamiana* (Fig. 1). But plants agroinfected with LS-MIM165/166 displayed symptoms including stunting and distortion of tissues at the top of the plants (Upper panel, Fig. 3a), including altered leaf shape, outgrow of leaf abaxial surface (enations) (Middle panel, Fig. 3a), and deformation of flowers (Lower panel, Fig. 3a). It is worth noting that in *N. benthamiana* plants the expression of an STTM for miR165/166 from a TRV-derived vector did not cause such a strong phenotype as seen here. Using TRV-STTM165/166 Sha and colleagues^16^ noted evidence for some loss of apical dominance and production of enations but not the same drastic effects seen with the CMV-delivered TM for miR165/166 (Fig. 3a).

Similarly, distinctive changes in leaf phenotype were also observed on tobacco plants infected with LS-MIM165/166 by agroinfection (Fig. 3b). Small leaves grew from the petioles of tobacco plants infected with LS-MIM165/166 (Fig. 3b), consistent with known roles of miR165/166 in regulation of meristematic activity^24^. Leaves were also distorted in shape with reduced lamina area and production of enations from vein tissue on the abaxial surface (Fig. 3) indicative of disruption of adaxial/abaxial and medial/lateral features of leaf development that occur in plants when miR165/166 activity is inhibited^24^. The control TM construct, LS-MIM-CK, caused no obvious symptoms or change of phenotype in tobacco (Fig. 3b). Taken together, the results obtained with *N. benthamiana* and tobacco plants indicate that the CMV-based TM expression is generally effective for *Nicotiana* species. We went on to investigate if the system would be effective in plants of another genus of the *Solanaceae* by agroinfection of tomato plants with LS-MIM165/166.

**Figure 4:**
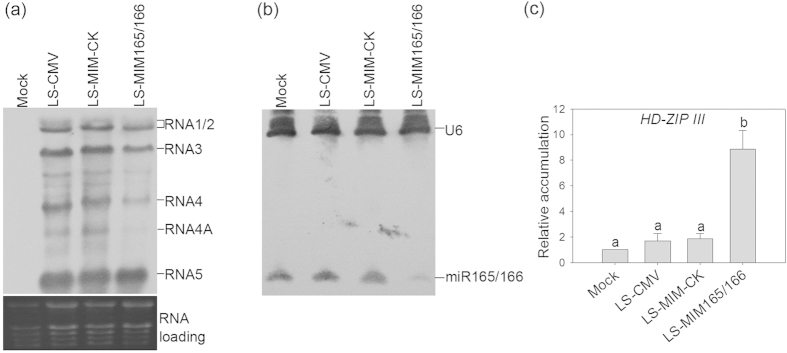
Accumulation of viral RNA, *miR165/166* and *HD-ZIP III* in tomato plants. Total RNA was extracted from non-inoculated compound leaves of mock-infiltrated plants or plants agroinfiltrated with *A. tumefaciens* cells carrying LS-CMV, LS-MIM-CK, or LS-MIM165/166 at 28 days post-infiltration. (**a**) Viral RNAs were separated by agarose gel electrophoresis, transferred to membranes and probed using a digoxigenin-labeled DNA oligonucleotide complementary to the sequence of the highly conserved 3′ un-translated region shared by of CMV genomic RNAs 1, 2, and 3 and by the sub-genomic viral RNAs 4, 4A and 5. Equal loading of lanes of the northern blot was checked by ethidium bromide staining revealing rRNA bands (lower panel). (**b**) Double-probed small RNA northern blot showing accumulation of miR165/166 and U6 RNA (loading control). (**c**) Results of reverse transcription-quantitative PCR for the miR165/166 target mRNA *HD-ZIP III*. The mean values were generated from three independent experiments. Different letters (**a,b**) indicate significant difference (ANOVA with post-hoc Tukey’s test, *P* < 0.05). Bars represent standard errors about the mean.

A previous report indicated that only mild symptoms (a small decrease in leaf blade size without any overall plant stunting) are induced in tomato (cv. UC82) by LS-CMV and that no alteration in accumulation of miR165/166 or its star strand occurs during infection with this strain^22^. Our observations of the effects of LS-CMV on the tomato variety Moneymaker are consistent with those of Cillo and colleagues^22^. At 28 dpi, tomato plants that had been agroinfected with LS-CMV or its TM control variant LS-MIM-CK displayed no stunting of growth and leaf morphology was indistinguishable from mock-inoculated plants (Fig. 3c). However, by 28 dpi tomato plants agroinfected with LS-MIM165/166 showed stunting (Fig. 3c, upper panel), remarkable deformation of upper compound leaves (Fig. 3c, middle panel), and leaflets exhibited growth of enations from the veins on the abaxial surface (Fig. 3c, lower panel) which, as discussed above, is a hallmark of disrupted miR165/166 activity^24^. In systemically infected leaves of agroinfiltrated plants, we found that steady-state levels of virus-specific RNAs were similar for LS-CMV, LS-MIM-CK, and LS-MIM165/166 (Fig. 4a). Consistent with the effects seen on plant phenotype, we found that steady-state accumulation of miR165/166 was depressed in leaves of plants infected with LS-MIM165/166 (Fig. 4b), whilst in these tissues the accumulation of the target mRNA *HD-ZIP III* was elevated (Fig. 4c). The levels of both of these RNA species were similar in mock-inoculated plants and plants agroinfected with LS-CMV or the TM control, LS-MIM-CK (Fig. 4).

In summary, the PCR-free cloning strategy allows simple, time- and cost-effective generation of constructs for 35S promoter-mediated transcription of CMV-derived TMV vectors for agroinfection. Infection with either LS-CMV or LS-MIM-CK caused no obvious disease symptoms in three solanaceous species. Furthermore, LS-MIM165/166 induced easily observable changes in phenotype, in contrast to the relatively mild changes induced by a TRV-derived vector carrying a corresponding STTM^16^. Taken together with the very wide host-range of CMV, the results suggest that TM vectors based on LS-CMV could prove especially useful for investigation of plant development in multiple species and the ease with which they can be generated and inoculated makes them potentially usable for high-throughput analyses of miRNA functions.

## Methods

### Plants

The plants used in this study were tobacco (*Nicotiana tabacum* L.) cv. Xanthi-nc, *N. benthamiana* (Domin.), and tomato (*Solanum lycopersicum* L. syn. *Lycopersicon esculentum* Mill.) cv. Moneymaker. Plants were grown under a 16-hour photoperiod with a light intensity of 150–200 μE.m^−2^.s^−1^ at 25 °C.

### Clone construction

We constructed infectious clones of LS-CMV genomic RNAs 1, 2 and 3 in which the virus-derived sequences are fused to a duplicated 35S promoter (Fig. 2a). Briefly, full-length cDNAs of LS-CMV RNA1, 2 and 3 were amplified from the DNA constructs pLS109, pLS209 and pLS309 described by Zhang *et al.*^23^. The amplified cDNAs of RNA1 and RNA2 were digested with *Bam*HI, and subsequently cloned into the plasmid pCB301 predigested by *Stu*I and *Bam*HI, to generate pCB301-LS109 and pCB301-LS209 respectively. The amplified cDNA of RNA3 was ligated into pCB301 pre-digested by *Stu*I and *Sma*I, to create pCB301-LS309. These cDNA clones were sequenced, and their infectivity was confirmed by infection of *N. benthamiana* via agroinfiltration (data not shown). Subsequently, cloning sites of *Spe*I and *Mlu*I were inserted between CP and 3′ UTR (un-translated region) in the infectious clone pCB301-LS309, to generate pCB301-LS309-S/M (Fig. 2b). The insertion of cloning sites was accomplished using two partially overlapped oligonucleotides oligo1 5′ AGACTAGTAGTACT*ACGCGT*TCCGTGTGTTTACCGGCGTC 3′ and oligo2 5′ GA*ACGCGT*AGTACTACTAGTCTAAGTCGGGAGCATCCG 3′ (the nucleotides underlined indicate *Spe*I, the italic nucleotides indicate *Mlu*I). The protocol for cloning TM sequences into pCB301-LS309-S/M is illustrated in Fig. 2b. Briefly, annealing two partially overlapped and completely complementary oligonucleotides, named Forward oligo and Reverse oligo were annealed to generate an oligonucleotide duplex with two sticky termini. Forward oligo carried target mimic sequence flanked with 5′ CTAGT and 3′ A, and Reverse oligo carried complementary sequence of target mimics flanked with 5′CGCGT and 3′ A. The resultant oligonucleotide duplex was ligated with the plasmid pCB301-LS309-S/M (pre-digested with *Spe*I and *Mlu*I) to create pCB301-LS309-MIM carrying a TM sequence. Using the protocol, we constructed pCB301-LS309-MIM165/166 and pCB301-LS309-MIM-CK by inserting the miR165/166 target mimic sequence (MIM165/166) and the microRNA-unrelated TM sequence (MIM-CK) adapted from the previous reports^13^,^19^. Constructs were authenticated by DNA sequencing prior to transformation of *Agrobacterium tumefaciens* GV3101.

### *In vitro* synthesis of viral RNAs and plant inoculation

Infectious clones of pFny109, pFny209 and pFny309 for Fny-CMV, and pLS109, pLS209 and pLS309 for LS-CMV have been described previously by Rizzo & Palukaitis^25^ and Zhang *et al.*^23^, respectively. A pLS309 variant, pLS309-MIM165/166 carrying a miR165/166 target mimic sequence (MIM165/166) was described previously^19^. *In vitro* transcription of infectious clones was carried out using mMESSAGE mMACHINE T7 transcription kits (Life Technologies). *N. benthamiana* plants at the five to six true leaf stage were infected by LS-CMV and LS-MIM165/166 by co-inoculating *in vitro*-synthesized transcripts of pLS109 and pLS209 together with that of pLS309 or pLS309-MIM165/166, respectively (mock mechanical inoculation used sterile water). Meanwhile, *N. benthamiana* plants were infected with Fny-CMV by inoculating *in vitro*-synthesized transcripts of pFny109, pFny209 and pFny309.

### Agroinfiltration

All T-DNA clones were separately introduced into *A. tumefaciens GV3101* by electroporation using a Gene Pulser (Bio-Rad). *A. tumefaciens* cells were grown in Luria-Bertani liquid medium with 50 μg/ml kanamycin and 20 μg/ml rifampin at 28 °C in a shaker. Cells carrying pCB301-LS109 and pCB301-LS209 were equally mixed with cells carrying pCB301-LS309, pCB301-LS309-MIM165/166 or pCB301-LS309-MIM-CK, and were adjusted to an optical density at 600 nm of 0.6 in infiltration solution [10 mM MgCl_2_, 100 mM acetosyringone and 10 mM 2-(N-morpholino) ethanesulphonic acid pH5.6]. Cell mixtures were infiltrated into the 4^th^ and 5^th^ true leaves of plants of *N. benthamiana* and tobacco at the five to six true leaf stage, and the cotyledons of tomato plants at the two true leaf stage. ‘Mock’ plants were infiltrated with infiltration solution containing no cells. The plants were photographed at approximately 4 weeks after agroinfiltration.

### RNA analyses

Upper, non-inoculated compound leaves of agroinfiltrated tomato plants were harvested at 28 dpi, and total RNA extracted using TRIzol (Life Technologies) according to the manufacturer’s instructions. RNA-gel blot (northern) analyses of CMV genomic RNAs and low molecular weight RNA were carried out as described previously^19^,^26^. Quantitative analysis of tomato miR165/166 target *HD-ZIP III* transcript steady-state accumulation was carried out using reverse transcription-quantitative PCR as described previously^10^ using primers for the tomato *HD-ZIP III* transcript described previously^27^. These experiments were carried out three times independently.

## Additional Information

**How to cite this article**: Liao, Q. *et al.* An improved cucumber mosaic virus-based vector for efficient decoying of plant microRNAs. *Sci. Rep.* **5**, 13178; doi: 10.1038/srep13178 (2015).
